# Elaborate design of shell component for manipulating the sustained release behavior from core–shell nanofibres

**DOI:** 10.1186/s12951-022-01463-0

**Published:** 2022-05-28

**Authors:** Yubo Liu, Xiaohong Chen, Yuhang Gao, Deng-Guang Yu, Ping Liu

**Affiliations:** 1 School of Materials and Chemistry, University of Shanghai for Science & Technology, 516 Jungong Road, Yangpu District, Shanghai, 200093 China; 2 Shanghai Engineering Technology Research Center for High-Performance Medical Device Materials, Shanghai, 200093 China

**Keywords:** Triaxial electrospinning nanotechnology, Core–shell nanofibres, Functionality, Polyethylene glycol, Sustained release, Hydrophilic

## Abstract

**Background:**

The diversified combination of nanostructure and material has received considerable attention from researchers to exploit advanced functional materials. In drug delivery systems, the hydrophilicity and sustained–release drug properties are in opposition. Thus, difficulties remain in the simultaneous improve sustained–release drug properties and increase the hydrophilicity of materials.

**Methods:**

In this work, we proposed a modified triaxial electrospinning strategy to fabricate functional core–shell fibres, which could elaborate design of shell component for manipulating the sustained-release drug. Cellulose acetate (CA) was designed as the main polymeric matrix, whereas polyethylene glycol (PEG) was added as a hydrophilic material in the middle layer. Cur, as a model drug, was stored in the inner layer.

**Results:**

Scanning electron microscopy (SEM) results and transmission electron microscopy (TEM) demonstrated that the cylindrical F2–F4 fibres had a clear core–shell structure. The model drug Cur in fibres was verified in an amorphous form during the X-ray diffraction (XRD) patterns, and Fourier transformed infrared spectroscopy (FTIR) results indicated good compatibility with the CA matrix. The water contact angle test showed that functional F2–F4 fibres had a high hydrophilic property in 120 s and the control sample F1 needed over 0.5 h to obtain hydrophilic property. In the initial stage of moisture intrusion into fibres, the quickly dissolved PEG component guided the water molecules and rapidly eroded the internal structure of functional fibres. The good hydrophilicity of F2–F4 fibres brought relatively excellent swelling rate around 4600%. Blank outer layer of functional F2 fibres with 1% PEG created an exciting opportunity for providing a 96 h sustained-release drug profile, while F3 and F4 fibres with over 3% PEG provided a 12 h modified drug release profile to eliminate tailing–off effect.

**Conclusion:**

Here, the functional F2–F4 fibres had been successfully produced by using the advanced modified triaxial electrospinning nanotechnology with different polymer matrices. The simple strategy in this work has remarkable potential to manipulate hydrophilicity and sustained release of drug carriers, meantime it can also enrich the preparation approaches of functional nanomaterials.

**Graphical Abstract:**

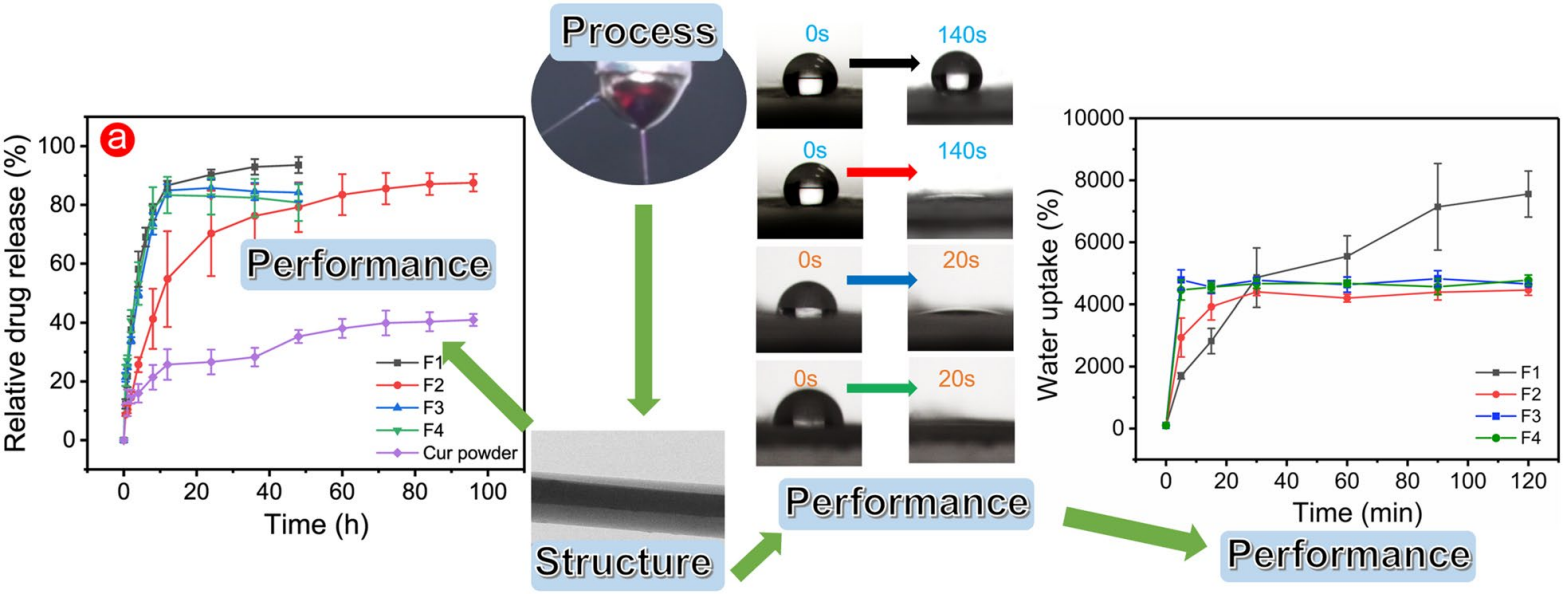

**Supplementary Information:**

The online version contains supplementary material available at 10.1186/s12951-022-01463-0.

## Background

Exploring the manufacture of functional materials has received considerable attention from many fields [1–5]. The functionality of materials always depends on their physicochemical properties [6–8]. Thus, these functional materials can be divided into electrochemical sensing materials [9], photosensitive materials [10], hydrophilic materials [11], antibacterial materials [12–14], and sustained-release drug materials [15–17]. The combination of different materials can theoretically improve functionality. However, the compatibility of physicochemical properties causes difficulties to form a tightly bonded material.

Electrospinning is a simple ‘top–down’ nanotechnology to prepare homogeneous functional materials, and the rapid fibre formation process is beneficial for the strong combination between materials [18–20]. Initial studies focused on monolithic mixed fibres containing 2 or 3 polymers by using the typical single-fluid electrospinning [21]. The incorporation of more polymers will undoubtedly improve the performance but is accompanied by weakened original functionality. The typical electrospinning remarkably limits the fabrication of fibres with improving two or more performance simultaneously. Fortunately, the development of advanced nanotechnology brings diversified electrospinning nanotechnologies, such as coaxial and triaxial electrospinning, to break this limitation, which leads to fabricating core–shell fibres [22]. Core–shell fibres provide a strategy that the two different polymers can form the unique layer, which can improve the performance simultaneously.

Building on the interests of core–shell fibres, coaxial and triaxial electrospinning methods have gradually replaced traditional single-fluid electrospinning. The fabrication of core–shell fibres has strict requirements on materials and usually needs spinnable materials. In coaxial electrospinning, the production of fibres needs good compatibility between the only two working fluids. Thus, the limited selection of different polymers makes the fabrication of core–shell fibres difficult. In standard triaxial electrospinning, all three working fluids are spinnable. The increased working fluids in triaxial electrospinning than those in coaxial electrospinning leads to more than twice the difficulty of fibre preparation. Fortunately, recent articles about unspinnable solvent facilitated the fibre preparation and provided remarkable inspiration for modified triaxial electrospinning [23–25]. Based on the above acknowledge, the three working fluids in triaxial electrospinning have an enormous potential to prepare core–shell fibres. The unspinnable solvent can be used as an outer working fluid to promote the preparation of functional fibres. Recent study indicated that this unspinnable solvent has a positive influence on the compatibility between the two other working fluids, thereby reducing the difficulty of preparing core–shell fibres [26]. Multiple-fluids electrospinning nanotechnology, as an advanced manufacturing process, promotes the development of intelligent, controllable, and efficient electrospinning nanotechnology [27, 28]. Zhao’s group prepared the functional fibres loaded with β-FeOOH/TiO_2_ by using a modified triaxial electrospinning process and showed that fibres could photodegrade 90.1% pollutants in water (doxycycline as a pollutant model) within 300 min, which was similar or even better than other powder photocatalysts [29].

In drug delivery systems, the fundamental properties of drug carriers directly affect the speed of drug release [30]. The rapid diffusion of water in hydrophilic materials always leads to a quick drug release especially for water-soluble materials, such as polyvinyl pyrrolidone (PVP), polyethylene glycol (PEG), and gelatin. The dissolution of these water-soluble drug matrices results in instantaneous and complete drug release. By contrast, water cannot enter hydrophobic materials easily. Thus, drugs are released at a slow rate. Over the past few decades, the drug matrix was often adjusted in accordance with the actual situation of patients. Hydrophilic materials are used to release drugs quickly in emergency situations, such as hemostasis and pain relief, and some chronic diseases need hydrophobic materials to provide a long-term drug supply. However, these single-performance drug matrices cannot be targeted for complex practical situations, such as exudate near chronic wounds. These matrices need a hydrophilic matrix to absorb exudate quickly and a sustained drug treatment. Although the combination of hydrophilic and sustained-release drug properties will theoretically have improved treatment efficiency, the two properties are antagonistic and contradictory. Selectively uniting the advantages of hydrophilicity and sustained-release drug in common drug carriers is difficult. The rate of drug release is highly correlated with the efficiency of water intrusion into the matrix [31]. Fortunately, the appearance of modified multi-fluids electrospinning provides separate locations for polymers. In this innovative nanotechnology, several polymeric working fluids were used to manufacture functional fibres with different structures through the unique homemade spinneret. Structural fibres exhibited more properties and potentials than traditional monolithic fibres in the controllable micro–nano field [32]. Modified coaxial and triaxial electrospinning, as important branches of multiple-fluids electrospinning, have no limitation on working fluids, and the unspinnable pure drug solution (without polymer) can be a special working fluid used in preparing functional fibres, which can prolong the time of sustained-release drug [33]. In this way, the two polymers can fully use the advantages in the prepared functional core–shell fibres.

Cellulose acetate (CA), as a derivative of green cellulose, is a common filament-forming polymer. Given its biocompatibility, nontoxicity, hydrophilicity, and water insolubility, CA has potential in many fields, such as pollutants removal from water [34–36], fluorescent sensor [37], and suppression of heat transfer [38]. In biomedical field, CA nanofibres have been explored as potential drug carriers for their fascinating nano-efforts and high drug-loading efficiency. Recently, many CA-based drug carriers have been exploited. Liu’s group prepared CA–zein monothetic fibres as efficient carriers for sesamol to provide a 1.5 h release profile in ethanol and a 48 h release profile in water [39]. Drug carriers with different structures provide space to store drugs, which can affect the drug release behavior. For instance, the Janus fibres can provide a two-stage controlled release profile [40]. For water-soluble polymers, PVP and PEG are used as common carriers for quick drug release and are added to increase the hydrophilicity of materials. Rashkov’s group suggested that the incorporation of hydrophilic PVP can increase the hydrophilicity of CA fibres and lead to a quick drug release [41].

In this work, we propose a simple modified triaxial electrospinning strategy to manipulate hydrophilicity and sustained-release drugs properties of functional nanomaterials. Compared with the traditional triaxial electrospinning, the unique outer unspinnable pure solvent positively influences the production of functional fibres. The model drug curcumin (Cur) is stored in the core CA matrix, and the CA matrix with water-soluble PEG is designed as the shell layer. The addition of PEG is designed to improve the hydrophilicity, and the whole shell layer (containing CA matrix and PEG) is designed as a protective layer to prevent drug release. Prepared functional fibres improved hydrophilicity and provided modified sustained-release drug profiles. This work explores the relationship between material properties and structure by using the modified triaxial electrospinning strategy.

## Materials and Methods

### Materials

CA white powder (acetyl group = 39.8 wt.%), PEG (*M*_w_ = 2000), and PVP K13–18 (*M*_w_ = 10,000) were purchased from Aladdin Reagent Co., Ltd. (Shanghai, China). PVP K30 (*M*_w_ = 50,000) was supplied by Sigma-Aldrich Trading Co., Ltd. (Shanghai, China). Cur, acetone, ethanol, N, N-dimethylformamide (DMF), Tween 80, and phosphate buffer saline (PBS) were purchased from Sinopharm Chemical Reagent Co., Ltd. (Shanghai, China). Methylene blue and magenta powders used for dyeing working fluids were purchased from Tianjin Zhiyuan Chemical Reagent Co., Ltd. (Tianjin, China). All chemical reagents were used directly without further purification.

### Triaxial electrospinning

**Fig. 1:**
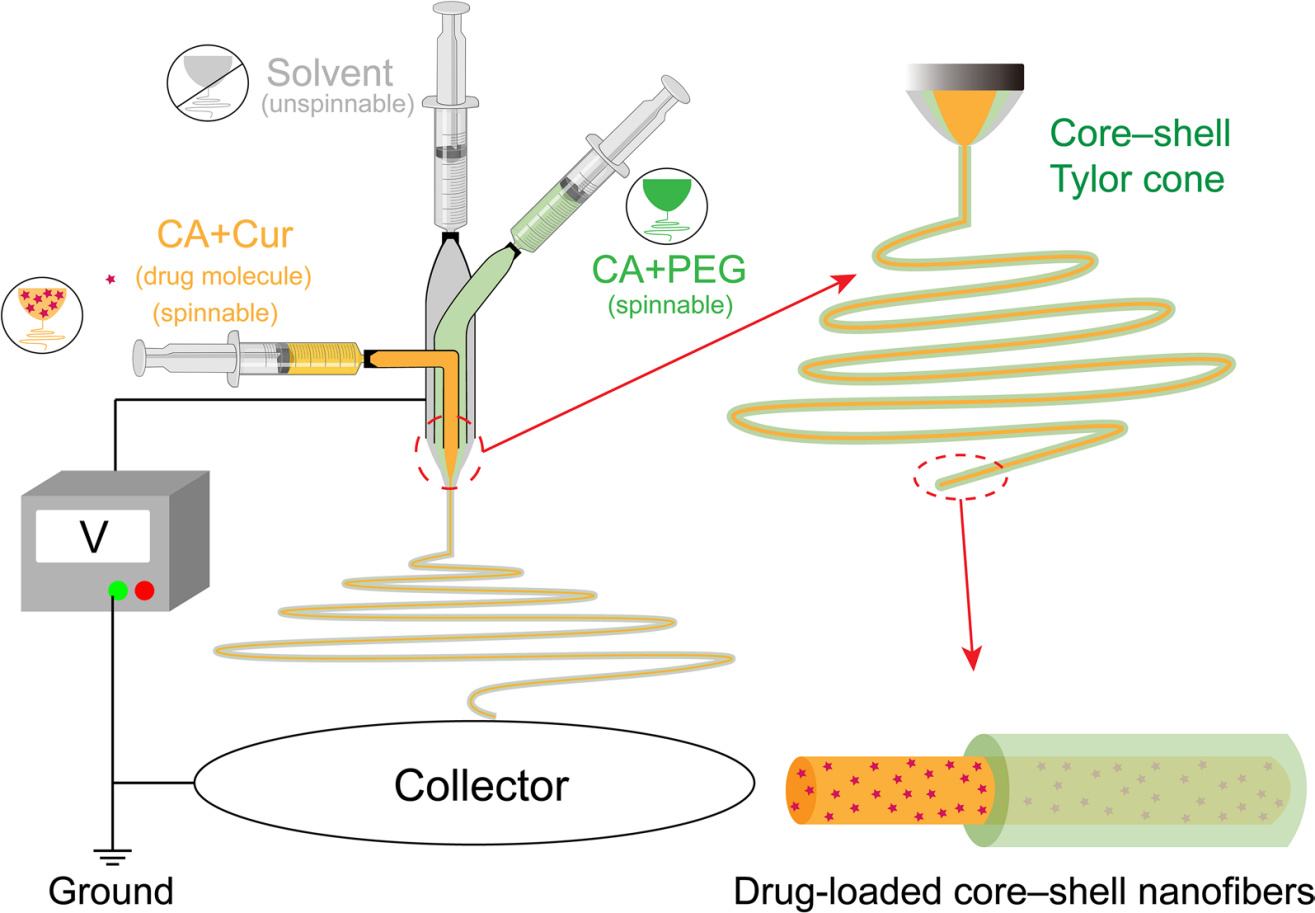
Schematic diagram of the modified triaxial electrospinning process and the drug-loaded core–shell nanofibres

In this work, CA was used as the main polymer, and PEG was added as a key hydrophilic polymer in relatively small amounts in special functional fibres. Table 1 provides the details of the fabrication of monolithic fibres F1 and functional core–sheath fibres F2–F4. The four fibres were prepared by solvent (unspinnable) circulation through modified multiple-fluids electrospinning process. As shown in Fig. 1, like the traditional single-fluid electrospinning, the modified triaxial electrospinning should be composed of four parts. The pump provided a constant driving force for the 10 ml syringe of working fluids. The combined action of the spinneret and high voltage forced the solvent evaporation and fabrication of the structural fibres, and the grounded aluminium foil was used to collect the produced fibres. Yunfan YFSP-T electrospinning instrument (Tianjin, China) was used to prepare the four electrospun fibres under approximately 10 kV.

**Table 1:** Details of the parameters for the four electrospun fibres

No	Working process	Applied voltage (kV)	Propelling speed of pump rate (mm/s)			Morphology	Diameter (nm)
			Inner^a^	Middle^b^	Outer^c^		
F1	Modified coaxial electrospinning	9.0	0.0014	-	0.0003	Straight fibres	500 ± 130
F2	Modified triaxial electrospinning	9.6	0.0014	0.0014	0.0014	fibres with few beads	540 ± 330
F3		10.2	0.0014	0.0014	0.0014	fibres with few beads	600 ± 260
F4		10.4	0.0014	0.0014	0.0014	fibres with few beads	520 ± 190

^a^ The inner working fluid contained 1.44 g CA and 0.24 g Cur was dissolving in 12 ml mixture of ethanol, acetone, and DMF (1:4:1, v:v:v).^b^ The middle working fluid contained 1.44 g CA and a certain amount of PEG (0.12 g in F2, 0.36 g in F3, and 0.72 g in F4) in 12 ml mixture solvents which were used the same in the inner working fluid. ^c^ The outer working fluid was composed of ethanol, acetone, and DMF (1:4:1, v:v:v).

## Characteristics

### Morphology and structure of the prepared fibres

The prepared fibres were observed by scanning electron microscopy (SEM, Florida, USA) at a voltage of 30 kV and working distance of 10 mm. Prior to observation, fibres should be sputtered with platinum in a nitrogen environment to confer its conductivity. The average diameter of at least 100 fibres in SEM was calculated by using the Image J software (Maryland, USA). Deionised water was dropped into the copper mesh to fix the few collected fibres, then transmission electron microscopy (TEM, Hitachi, Japan) was used to observe the internal structure of fibres after drying.

### Physical and compatibility analyses

Physical forms of powders and fibres were tested using the MiniFlex 600 (Rigaku, Japan), and flattened samples were conducted from 10° to 70° at a rate of 5° min^–1^. All samples were analysed using Fourier transform infrared (FTIR, PerkinElmer, Billerica, USA). The test was conducted at a range of 500–4000 cm^–1^, and samples were scanned eight times.

### Water contact angle and water uptake analyses

Fibre mats were cut into 20 mm × 60 mm rectangles and glued onto the glass slide smoothly. The JC2000C1 interface tension measuring instrument (Shanghai, China) was used to conduct the water contact angle tests of prepared rectangle samples. Each sample needed to drop at least six droplets, and the angle was calculated using the ImageJ software (Maryland, USA).

Dry fibres (0.02 g) were placed into the 500 ml PBS (37 ℃, pH = 6.8) at a constant rate of 50 rpm. Placed the wet fibres in a dry beaker and recorded the weight. Samples were tested at least three times and results were recorded as mean ± standard deviation (SD). The water uptake efficiency was calculated using Eq. (1):

1 $$W \left(\%\right)=\frac{{W}_{wet}-{W}_{dry}}{{W}_{dry}}\times 100\%,$$

where *W* is the water uptake efficiency, *W*_*wet*_ is the weight of wet fibres at each time interval, and *W*_*dry*_ is the weight of initial dry fibres.

The hydrophilic properties of prepared fibres were evaluated via the following equations.

2 $${\gamma }_{LG}cos{\theta }_{C}={\gamma }_{SG}-{\gamma }_{SL},$$

where ${\theta }_{C}$ is the equilibrium water contact angle, ${\gamma }_{LG}$ is the interfacial energy of liquid–vapor, ${\gamma }_{SG}$ is the interfacial energy of solid–vapor, ${\gamma }_{SL}$ is the interfacial energy of solid–liquid. When a drop of water on a solid surface is subjected to strong interfacial force (strongly hydrophilic solid surface), the water should be completely flat on the solid surface with a contact angle of 0°. Other hydrophilic solid surfaces often have a contact angle ranging between 0° to 90° (water contact angle on many high hydrophilic surfaces is from 0° to 30°). In contrast, the hydrophobic solid surfaces often show a contact angle larger than 90°.

### In vitro drug dissolution

The drug dissolution test was conducted in accordance with the pulp method in the Chinese Pharmacopoeia (2015 version). Drug-loading fibres (20 mg) were placed in conical flasks with 200 ml PBS (0.5% [*v/v*] Tween 80, pH 6.8), and flasks were fixed in a shaking incubator (SHA-AB, Jiangsu, China) at 37.5 °C with a constant shaking rate of 50 rpm. The solution (4 ml) was collected at set intervals and added with an equal fresh PBS with 0.5% (*v/v*) Tween 80. The absorption of the obtained solutions was obtained using a UV–vis spectrophotometer (UV-2102PC, Shanghai, China) at ${\lambda }_{max}=425 nm$, and the absorption data could be transferred to the drug cumulative release based on the standard curve.

Drug-loaded fibres (20 mg) were dissolved into acetone, and the solution was added with PBS (0.5% [*v/v*] Tween 80) up to 200 ml. The total amount of drug in fibre was calculated on the basis of 4 ml in the final homogeneous solution. All results were expressed as mean ± SD and *n* = 3. The drug loading efficiency could be calculated by Eq. (3):

3 $$E \left(\%\right)=\frac{{M}_{Actual}}{{M}_{\mathrm{Theoretical }}}\times 100\%,$$

where *M*_*Actual*_ is the actual measured total amount of drug, and *M*_*Theoretical*_ is the theoretical amount of drug in working fluids.

Drug release data could be further processed using mathematical models. The first-order model was used to quantify the drug release data, and the Peppas model was used to evaluate the drug release mechanisms.

4 $$F = F_{0} ({1 } - {\text{ e}}^{ - kt} ),{\text{and}}$$

5 $$Q = kt^{n} ,$$

where *F* and *Q* are the cumulative drug releases at time *t*, *F*_0_ indicates the initial drug amount in the first-order model, *k* is the release rate constant, and *n* is the characteristic parameters in drug diffusion.

## Results and discussion

### Triaxial electrospinning process

**Fig. 2:**
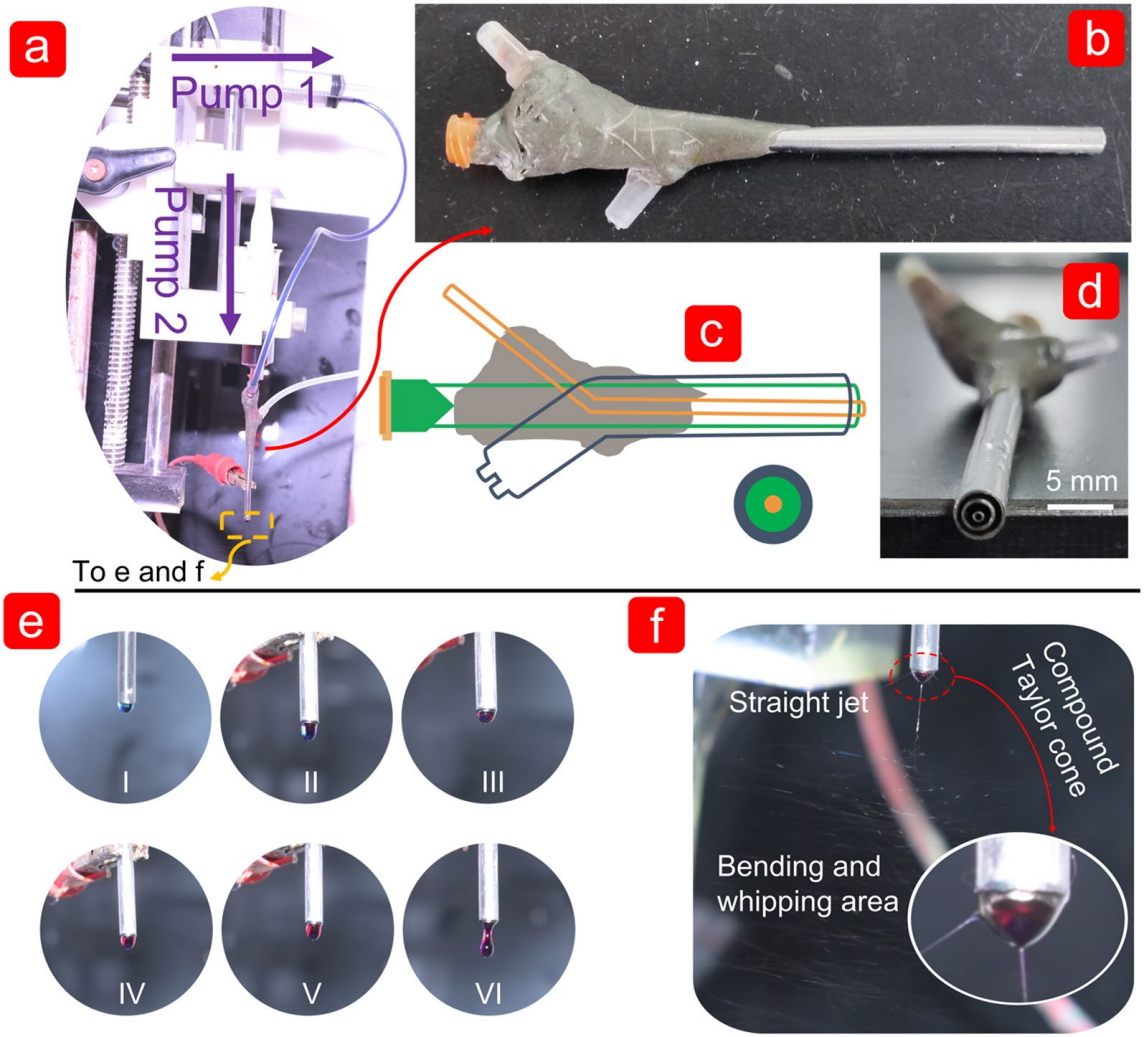
Digital photos of the implementation of the triaxial electrospinning process after dyeing. **a** The implementation of electrospinning process including pumps, syringe, silicone tube, homemade spinneret, and alligator clip (provide high voltage); **b**, **c** were the external appearance and internal structure of the homemade spinneret, respectively; (d) concentric structure of the homemade spinneret; **e** naturally dropping of working fluids without voltage applied; **f** the manufacturing process of triaxial electrospun fibres including Taylor cone, straight jet, and bending and whipping area

The clear preparation process of the triaxial electrospun fibre was based on the dyeing of the working fluids. The inner working fluid was dyed dark blue by methylene blue, whereas the middle working fluid was dyed red by magenta. In this work, inner and outer working fluids were driven by the same lateral driving force, and the middle working fluid had a vertical driving force due to the internal structure of the homemade spinneret (Fig. 2a–d). The vertical collection method was designed to facilitate the observation and preparation of fibres. Similarly, the F1 fibres prepared by modified coaxial electrospinning also used this method (Additional file 1: Fig. S1). The inner working fluid was dyed dark red by magenta, whereas the outer working fluid was transparent. Although a small amount of dye was diffused into the transparent sheath, the core–shell structure could still be distinguished by the shade of colour. For the modified triaxial electrospinning process, Fig. 2e shows the compound droplets with different colours around the homemade spinneret caused by different driving forces. At the beginning stage, the blue liquid was surrounded by a transparent liquid in Fig. 2e (I) under the driven of inner and outer working fluids. The red dyeing fluid gradually appeared in the middle of the droplet driven by middle working fluid. Increased driven speed of the middle working fluid resulted in red area in the droplet. In Fig. 2e(II–VI), the compound droplet exhibited a transparent–red–blue structure from outside to inside, including the dripping of the droplet.

Figure 2f showed a clear fabrication process of the electrospun triaxial fibres, where the voltage was added after the three colors appeared in the droplet. The three main stages in the fabrication process of fibres included compound Taylor cone, straight jet area, and a complex circle whipping area. The whole process exhibited a magic phenomenon of the preparation of multi-layers fibres in a short time. The partial zoom of the compound Taylor cone in the right corner of Fig. 2f, including a blue inner layer, a red middle layer, and a transparent outer layer.

### Surface morphology and the internal structure of fabricated fibres

**Fig. 3:**
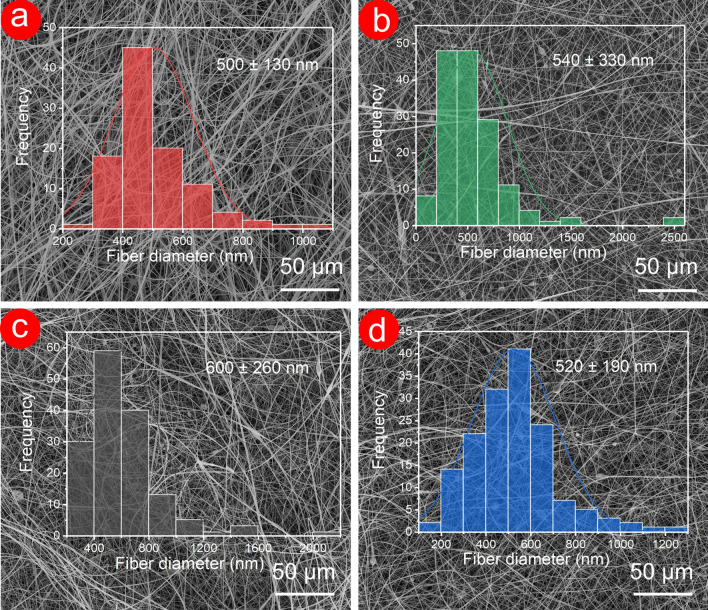
SEM images and average diameter distribution of F1 (**a**), F2 (**b**), F3 (**c**), and F4 (**d**) fibres

The microscopic surface morphology of fabricated fibres is shown in Fig. 3. F1 fibres exhibited a uniform and smooth cylindrical surface with an average diameter of 500 ± 130 nm. By contrast, F2–F4 fibres prepared by modified triaxial electrospinning did not have perfect morphology and showed a cylindrical shape with a certain number of bead-on-a-string fibres. The average diameter of F2–F4 fibres was slightly increased compared with that of F1 fibres, which implied the formation of a double–layer structure. Few beads-on-a-string morphologies in F2–F4 fibres could contribute to the PEG component, which could not be optimized by using the modified triaxial electrospinning. Meanwhile, the average diameter of F2–F4 had no significant connection with the PEG content from SEM images.

**Fig. 4:**
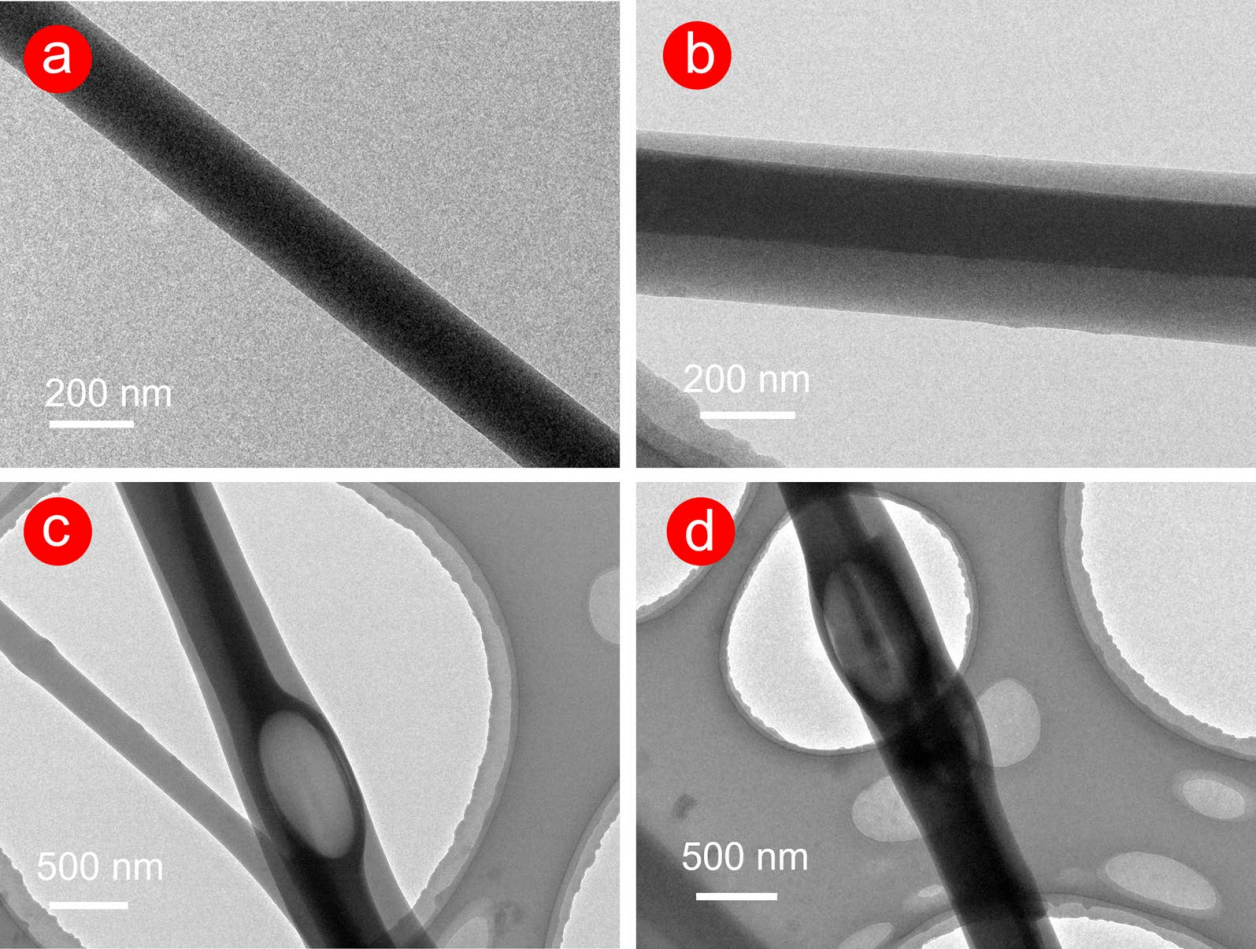
TEM images of F1 (**a**) and F2–F4 (**b**–**d**) fibres

As shown in Fig. 4, the internal structure of the fabricated fibres was observed clearly by TEM. F1 fibres exhibited a straight and single-layer structure, while the core–shell structure of F2–F4 fibres was apparent. However, the core layer was not exactly in the centre of the fibre in Fig. 4b–d. This result was probably attributed to the randomness of the preparation of the fibres in the electrospinning process. As shown in Fig. 4c, d, the fibre with beads-on-a-string morphology exhibited a core–shell ‘bubble’ structure, which was closely related to the electrospinning process. Linear, spindles/beads-on-a-string, and particle morphology produced in the electrospinning process were normal, which were inseparable from the elasticity and viscosity of the working fluid. In this work, the incorporation of the PEG component may decrease the viscosity of F2–F4 working fluids. The rapid stretching effect of electrostatic action on F2-F4 working fluid is reduced during the bending and whipping area in Fig. 2f. Thus, the incomplete stretching of the working fluid under the electric field leads to linear with beads-on-a-string morphology (‘bubble’ structure).

The different greyscales in black and white TEM images suggested the structural changes, and the grey level could be influenced by thickness, density, and elements [42]. In this work, CA was the principal component in the two prepared fibres, and the PEG component in F2–F4 could be ignored due to the few contents. Thus, density and thickness were the key factors that led to the grey level. In Fig. 4b–d, 2% drugs were stored in the core of F2–F4 fibres, which increased the molecular entanglement of the CA polymeric matrix to a certain degree, thus increasing the core density of F2–F4 fibres. In addition, based on the top–down observation method in the TEM, the grey level of the core layer was composed of the original core layer and the part of the sheath layer, which led to a ‘thick’ core layer. Therefore, the synergy of density and thickness led to a high grey level of core in Fig. 4b–d.

### XRD and FTIR

**Fig. 5:**
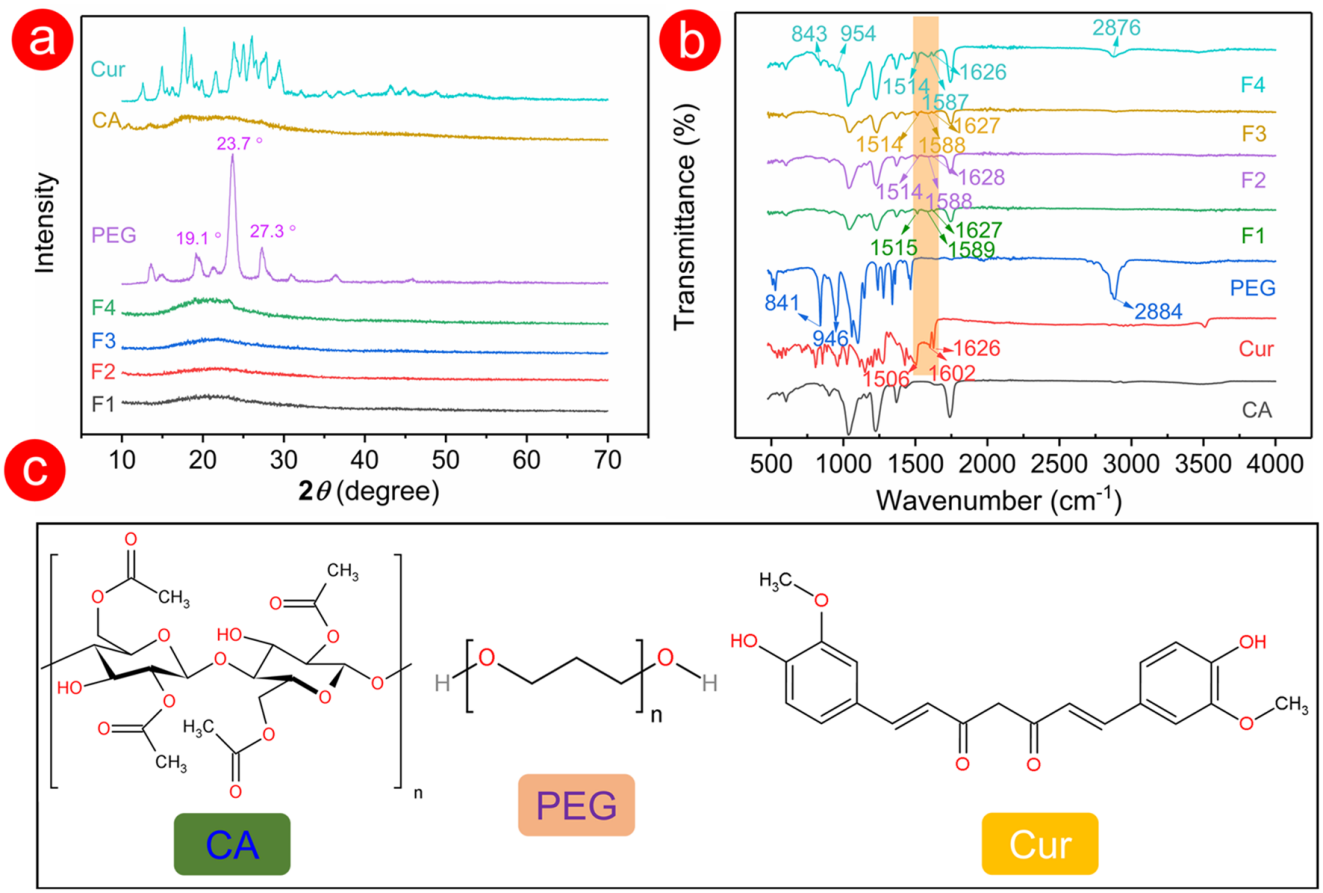
The XRD pattern **a** and the FTIR spectrum **b** of the raw materials (i.e., PEG, CA, and Cur powders) and fibres. **c** Chemical structure of the raw materials

In Fig. 5a, a small number of short peaks appeared in the X-ray diffraction (XRD) pattern of CA powder (the main component in fibres), which suggested that CA powder had a certain crystallinity. However, these early short peaks did not appear in the XRD pattern of the four fabricated fibres in Fig. 5a. The CA component was evenly distributed after the process of electrospinning, and the substance with a certain degree of crystallinity no longer existed. In the XRD pattern of PEG, the highest peak shown at 23.7° represented the (112) plane, and peaks around 19.1° and 27.3° were assigned to the (120) and (222) planes, respectively. These sharp and other low intense peaks indicated that PEG was a typical crystal material [43–45]. For F1–F3 fibres, only one hump appeared in the corresponding XRD patterns, indicating amorphous forms. The low PEG component had no negative effects on the physical form of F2 and F3 fibres, but the XRD pattern of F4 fibre containing 6% (*w/v*) PEG showed a faint peak near 13–25°. For Cur, a series of sharp peaks also responded to typical crystal materials. By contrast, F1–F4 fibres had no characteristic peaks in their XRD patterns, which suggested that the physical form of drug crystal changed after the electrospinning process (from a crystalline state to an amorphous state). Electrospinning can be regarded as a physical drying process, where working fluids are converted into solid fibres during solvent evaporation. Prior to electrospinning, all working fluids were homogeneous transparent solutions. Drug molecules were highly dispersive to the surface of the solid micro/nanofibre in a short time during the electrostatic drying process and the crystal structure of the drug transferred to an amorphous state. Although the drug concentration in the fibre increased, no sharp peaks appeared in the XRD pattern of the drug (Additional file 1: Fig. S2). The electrospinning process is a reliable process to transform the drug from a crystalline to an amorphous state.

The FTIR spectra of raw materials (i.e., PEG, CA, and Cur) and fibres are shown in Fig. 5b. For PEG, the characteristic peaks at 2884 cm^–1^ indicated the asymmetric bending vibration of methylene groups (-CH_2_) [46]. In addition, the characteristic absorption bands at 946 and 841 cm^–1^ were related to the asymmetric stretching vibration of ether groups (-C-O-) [44, 47]. Although characteristic peaks of PEG did not appear in the spectrum of F2 and F3 fibres, a higher PEG content in F4 fibres showed clear characteristic peaks at 2876 cm^–1^ in the FTIR spectrum. Cur exhibited a carbonyl (C = O) at 1626 and 1602 cm^–1^ and olefins (-C = C-) at 1506 cm^–1^ [48–50]. F1–F4 fibres had the characteristic peaks of carbonyl and olefins of Cur, which proved that Cur had been successfully encapsulated. In addition, hydroxy groups (PEG) and free carbonyl groups (Cur and CA) would form the hydrogen bond to increase the compatibility in fibres (Fig. 5c), such as providing a stable exist for drug molecules in fibres.

### Water contact angle and swelling performance

The hydrophilicity of the drug-loaded fibres conducted by the water contact angle test is a key point in a rapid drug delivery to the desired site of the patient. The first research between water contact angle and interfacial tension of the drops on a plane was proposed by Thomas Young in 1805 [51]. Prior to preparing the functional hydrophilic materials, three hydrophilic polymers (i.e., PVP K13–18, PVP K30, and PEG) were added to the CA working solution. The addition of PVP hardly affected the hydrophilic properties of fibres, but the PEG component almost instantaneously improved the hydrophilicity of CA (Additional file 1: Fig. S3). The surface morphology of the fibres after adding hydrophilic ingredients hardly changed before and after water intrusion (Additional file 1: Fig. S4). This result indicated that the added agents PEG and CA polymeric matrix formed uniform fibres after the electrospinning process. In other words, the PEG component was evenly distributed in the fibres and did not form the pore structure after the water invade. The hydrophilicity of CA drug carriers could be adjusted by controlling the content of PEG. When the PEG content reached 3%, the water molecule was ‘absorbed’ by fibres in a short time. Thus, fibres showed a highly hydrophilic behavior. However, when the PEG content was reduced to 1%, fibres exhibited a hydrophobic state in the first 10 s (water contact angle > 90°), and fibres instantly changed from hydrophobic to highly hydrophilic after 10 s (Additional file 1: Fig. S5).

**Fig. 6:**
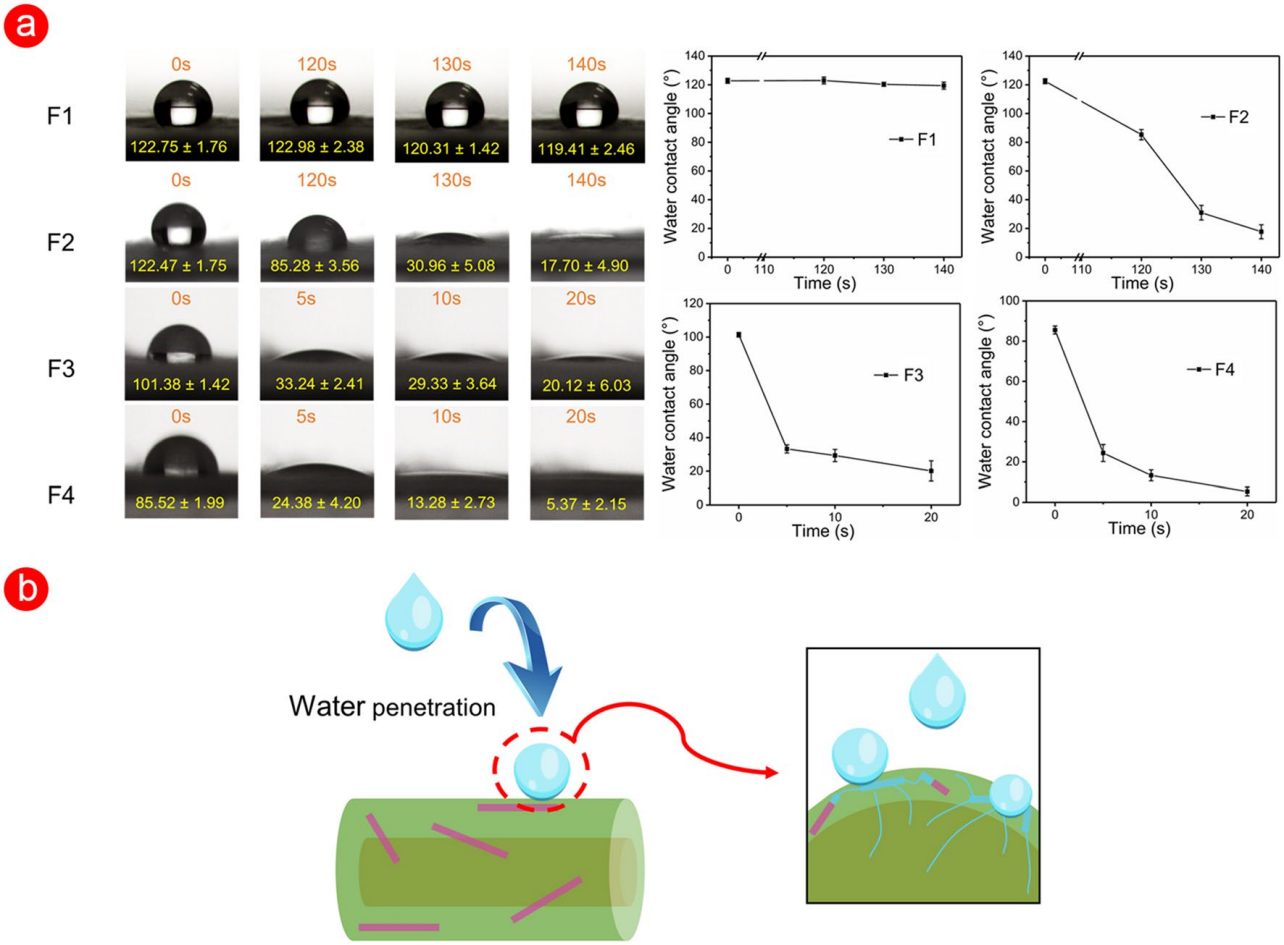
The surface wetting performance of fibres. **a** The water contact angle and its dynamic visualization of prepared fibres. **b** The improved hydrophilic mechanism of fibres

Triaxial electrospinning was used to fabricate functional F2–F4 fibres on the basis of the above acknowledge, where the CA polymeric containing PEG was designed as a middle layer to improve the hydrophilicity. In Figs. 6a, the water contact angle of F1 fibres was kept around 120° in 140 s, as a stable hydrophobic state. As time went by, the hydrophilic property of F1 fibres was enhanced, where the water droplet was gradually ‘absorbed’ by F1 (Additional file 1: Fig. S6). By contrast, F2–F4 fibres showed a unique dynamic water contact angle process in Fig. 6a. With the increase of content of PEG in core–shell fibres, the short–term hydrophilicity of the fibres was greatly improved. For F2 fibres, the water droplet was smaller at 120 s than that of the original state (from 122.47° ± 1.75° to 85.28° ± 3.56°). In the following 20 s, F2 fibres gradually exhibited a highly hydrophilic state. For F3 and F4 fibres, the time needed for this process was less than 5 s, and F4 with higher PEG content exhibited a water contact angle approaching 0° (5.37° ± 2.15°) than 20.12° ± 6.03° of F3 fibres. Compared with those of F1 fibres, the dynamic water contact angle images of F2–F4 fibres showed a transition from hydrophobic to hydrophilic in a short time, and the time could be manipulated by amount of PEG.

In Fig. 6b, a small amount of hydrophilic PEG in the fibres ‘absorbed’ the water molecules for the first time when the water came into contact with the surface of fibres. The molecular micropores left by dissolved PEG component formed a ‘passage’ in a short time to guide the water flow inward, which accelerated the process of invading the fibre matrix. Thus, water droplets decreased in size over time. The good hydrophilicity resulting from the combination of materials and structure will affect its other functionality, such as swelling properties and drug release properties of F2–F4 fibres.

**Fig. 7:**
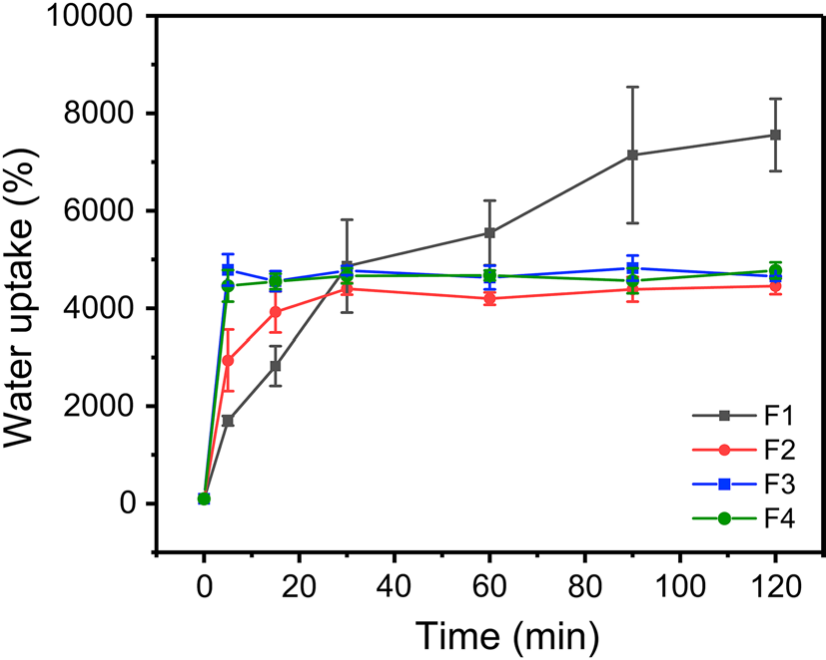
Swelling properties of F1–F4 fibres in 120 min

Figure 7 showed a dynamic water uptake efficiency of the prepared four fibres. F2–F4 fibres, prepared by modified triaxial electrospinning, had better water storage capacity than F1 fibres in the first 20 min. Specially, F3 and F4 fibres only needed 5 min to reach saturation, while F2 fibres needed 0.5 h. The increased PEG content had improved the water uptake efficiency of fibres at the early stage, which was similar to water contact angle results. By contrast, F1 fibres could absorb water at a considerable rate until 90 min, then stabilised at 120 min (near 7555%). The lower water uptake efficiency of F2–F4 than that of F1 suggested that the ‘passage’ left by the dissolved PEG component caused the water loss to a certain extent. Gap left by the dissolution of PEG reduced the water–holding capacity of functional F2–F4 fibres. The macrophotographs before and after the experiment (Additional file 1: Fig. S7) were collected to analyse the distinct swelling phenomenon of F1 and F2 fibres. F1 fibres were ‘fluffier’ and filled with many thin fibres. F2 fibres were relatively ‘dense’, in other words, their density was relatively high, which was similar to TEM results. In addition, F2–F4 left less fibrous residue on the aluminum foil than F1 (Additional file 1: Fig. S8). This result contributed to the core–shell structure produced using triaxial electrospinning, which led an increase in the bonding force between fibres. Thus, F2–F4 fibres could be easily removed from the aluminium foil. ‘Fluffy’ F1 fibres provided a strong ability to store enough water, whereas ‘dense’ (not enough space) F2–F4 fibres led strong shrinking phenomenon.

Moreover, the fibres’ swelling properties between recent articles and this work were compared (Table 2). Functional F2–F4 fibres had similar or even better swelling efficiency compared with other fibres as we know. In this work, although the swelling efficiency of F2–F4 fibres were weaker than that of F1 after 2 h, the initial ability of water uptake had remarkable potential in special situations, such as producing novel drug delivery carriers.

**Table 2:** Comparison of swelling properties of electrospun fibres in recent yea

Composition of the fibres	Process^b^	Immersion medium	Swelling capacity	References
10% PHBV	Single	PBS	320% (6 h)	[52]
12% PCL;	Single	PBS	86% (4 h)	[53]
10% PCL + 2% CS;	Single	PBS	103% (4 h)	
10% PCL + 2% CS 0.2% HA + 5% PEO	Single (layer by layer)	PBS	135% (4 h)	
10% PU	Single	PBS	459% (24 h)	[54]
7% PU + 5.1% CA		PBS	1090% (24 h)	
5% PU + 8.5% CA		PBS	1864% (24 h)	
3% PU + 11.9% CA		PBS	3153% (24 h)	
15% PCL	Single	PBS	328% (24 h)	[55]
15% PCL, 15% Gel	Double nozzle	PBS	406% (24 h)	
10% PCL	Single	Distilled water	74% (48 h)	[56]
8% PLA		Distilled water	225% (48 h)	
12% PCL/PLA		Distilled water	172% (48 h)	
F1	Coaxial	PBS	7555% (2 h)	This work
F2	Triaxial	PBS	4456% (2 h)	
F3	Triaxial	PBS	4654% (2 h)	
F4	Triaxial	PBS	4775% (2 h)	

*PHBV* Poly (3-hydroxy butyric acid-co-3-hydroxy valeric acid), *PCL* Polycaprolactone, *CS* Chitosan, *HA* Hyaluronic acid, *PEO* Polyethylene oxide, *PU* Polyurethane, *CA* Cellulose acetate, *Gel* Gelatin, *PLA* Polylactic acid. ^b^ Single, double nozzle, and triaxial denote single electrospinning, double-nozzle electrospinning, and triaxial electrospinning, respectively.

### Drug dissolution

**Fig. 8:**
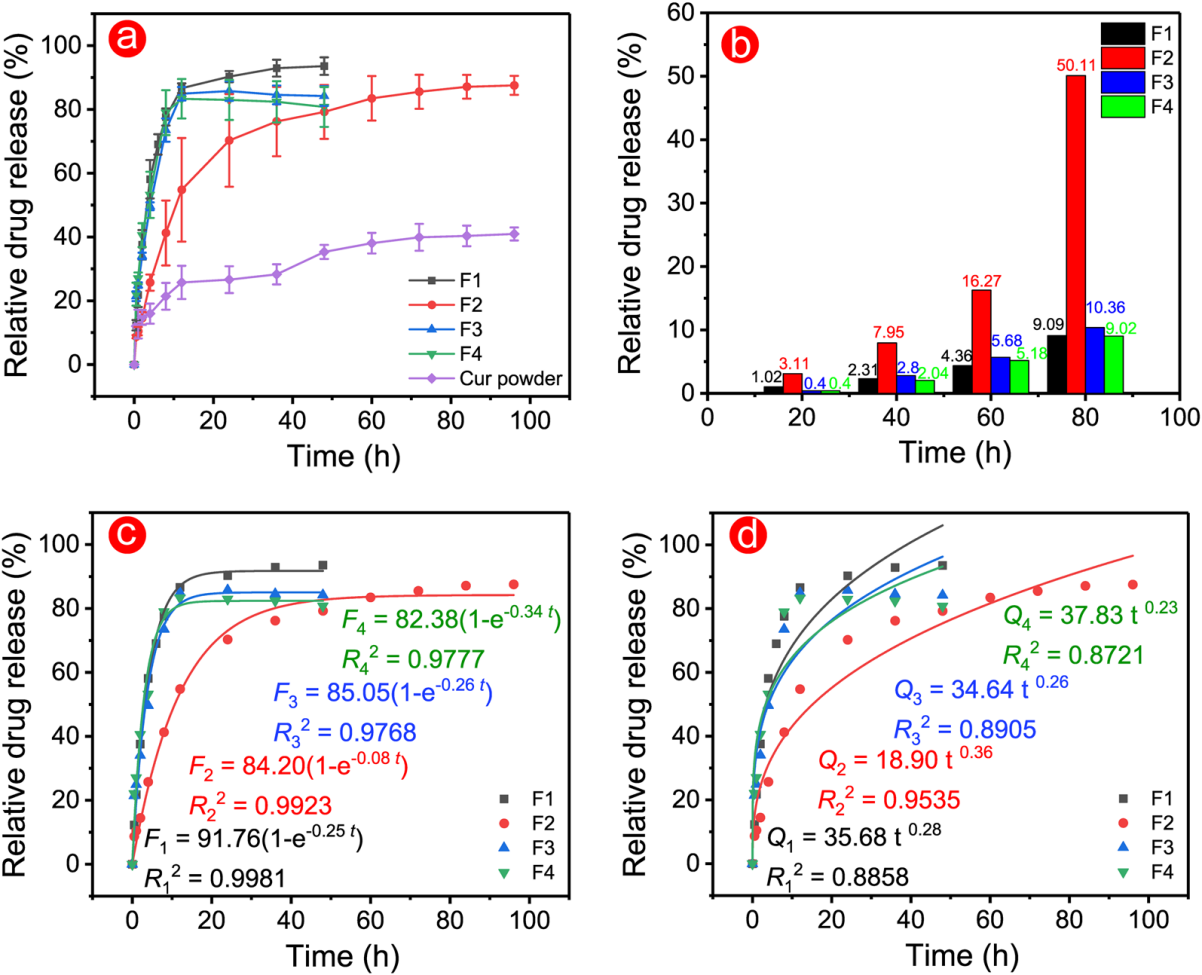
Drug dissolution tests of F1–F4 fibres. **a** Relative release profiles of fibres and Cur powder. **b** Relationship between time and relative release amount. Fitting results of the first-order model **c** and Peppas model **d** for drug release data

The electrospun fibre was the high-efficiency drug-loaded carrier [57, 58]. In this work, the calculated drug loading efficiencies of F1–F4 fibres were 100.79% ± 0.55%, 96.81% ± 4.45%, 93.16% ± 5.10%, and 92.09% ± 3.04, respectively. The high loading efficiency was due to the electrospinning nanotechnology and CA polymer. CA was proven to be an excellent biomaterial in drug delivery systems in recent decades [59–61]. The good hydrophilic and undissolved properties of CA brought a slightly longer drug dissolution behaviour. In Fig. 8a, monolithic F1 fibres had a 48 h drug dissolution profile, whereas functional F2 fibres had a more than 96 h profile. The middle blank layer in the triaxial electrospinning process provided a ‘barrier’ to prevent the process of drug release from inside out and prolonged the drug release time. However, this blank layer lost its ‘barrier’ effect in F3 and F4 fibres due to the high PEG content (over 3%), drugs were completely released in 12 h. Tailing–off effect is inevitable for drug carriers at the late drug release stage and is useless for patients. Fortunately, F3 and F4 fibres had improved drug release profiles, which eliminated the tailing–off effect and made fully use of drugs. The dissolution profile of Cur powders was irregular, and only 40.92% ± 2.03% of drugs were dissolved after the long term shaking time. The total drug release from the four fibres were 93.57% ± 2.74%, 87.54% ± 2.98%, 84.21% ± 3.10%, and 80.76% ± 6.24%, respectively. High drug dissolution suggested that electrospun fibres could improve and prolong these kinds of water-insoluble drugs. The low total drug dissolution of F2–F4 fibres could be attributed to the ‘barrier’ effect of the core–shell structure, which prevented the diffusion of the last few amounts of drugs.

Figure 8b shows the relationship between time and relative drug release. F1, F3, and F4 fibres exhibited a similar time required to reach each release stage, while F2 needed a long time period. With increased drugs released, the gap between the time required for F2 and other fibres was widened. In other words, the long time needed to reach the set release amount meant a slow drug release. These statistics provided direct evidence to demonstrate the improved sustained–release properties of functional F2 fibres with 1% PEG.

First-order and Peppas models were established to evaluate the drug release mechanisms in Fig. 8c and 8d [62–64]. All fibres were perfected following the first-order model due to good regression results (*R*_1_^2^ = 0.9981, *R*_2_^2^ = 0.9923, *R*_3_^2^ = 0.9768, and *R*_4_^2^ = 0.9777). The value of *n* in the Peppas model is the key parameter for evaluating release mechanisms. *n* values in formulas were less than 0.5, which indicated that drug release was dominated by the Fickian diffusion. Thus, all fibres had similar diffusion mechanisms. In addition, the *R*_2_^2^ = 0.9535 of F2 was higher than that of other fibres (*R*_1_^2^ = 0.8858, *R*_2_^2^ = 0.8905, and *R*_3_^2^ = 0.8721), which suggested that the regression result of F2 was better than that of other fibres. The above results reflected that a suitable combination of structure and materials had a positive effect on the drug release mechanisms, such as the suitable additive of hydrophilic PEG and the core–shell structure in this work.

### Drug release mechanism

**Fig. 9:**
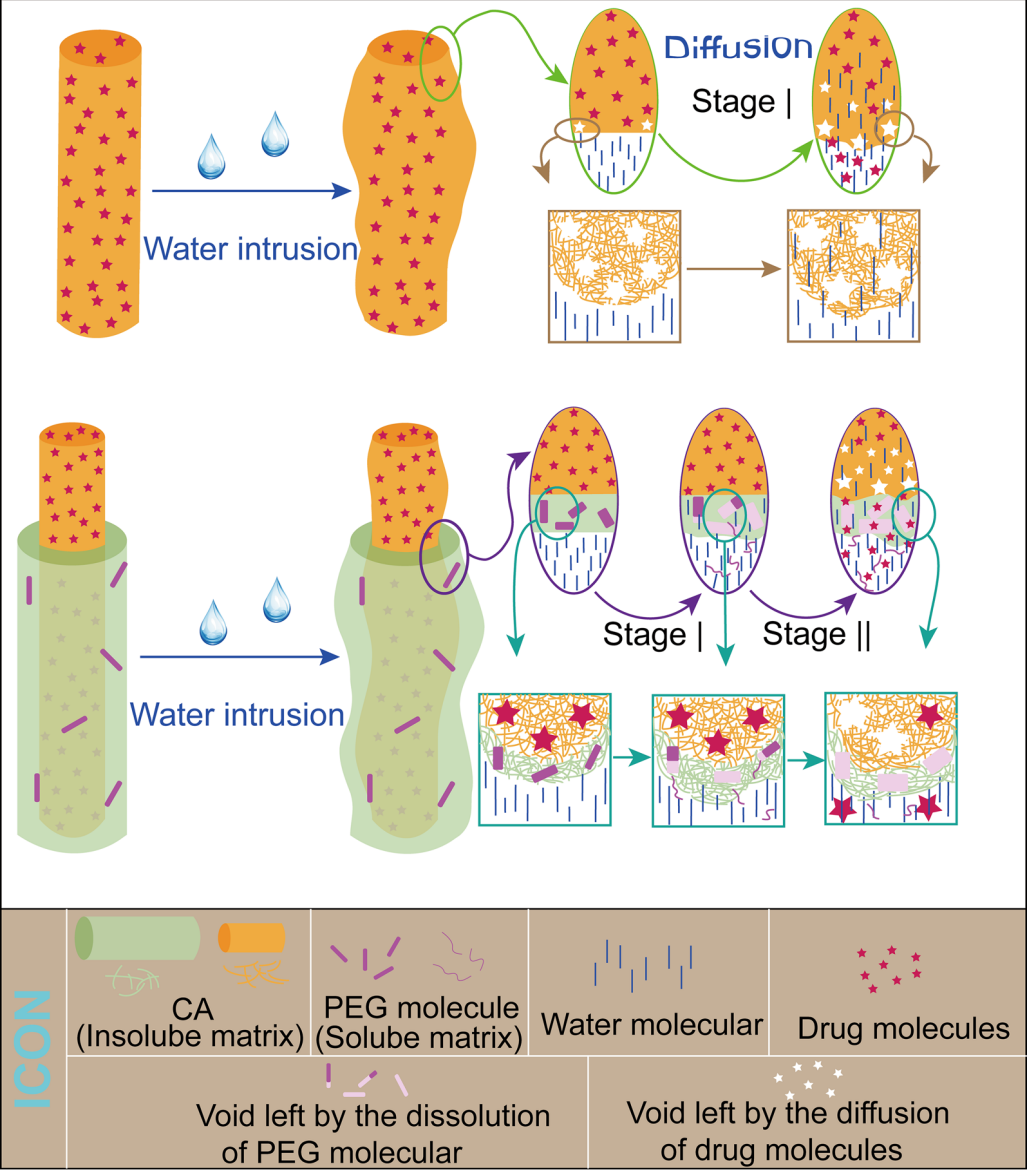
Mechanism of drug release from prepared fibres

The drug mechanisms of fibres were predominantly affected by polymeric properties (such as hydrophilic property) and structure. CA, as a natural swelling material after ‘absorbing’ water, has a high water-uptake performance that is beneficial for drug release. For monolithic F1 fibres in Fig. 9, drug molecules were diffused by water molecules into the release medium when they invaded the polymeric matrix, and the drug concentration difference between inside and outside the fibres was directly proportional to the drug release rate. The molecular gap left by drug molecular increased with the swelling of CA, and the high remaining water efficiency of drug carriers F1 provided favorable conditions for drug diffusion into the outside release medium. By contrast, the drug release mechanism of F2–F4 was complicated. Generally, the wettability of drug carriers had a remarkable influence on drug release, and excellent wettability always leads to a rapid drug release process. The incorporation of PEG had improved the hydrophilicity of functional F2–F4 fibres and the hydrophilicity improvement effect was significant when the content reached 3%. Thus, F3 and F4 had a quicker drug release that F2. Compared with the relatively quick release of F1, the continuous sustained-release process of F2 in the later stage could be attributed to the structural design and PEG component of fibres. Although the PEG component was dissolved as the water entered, the water-insoluble CA matrix extended the distance of water molecules to the core layer, as shown in Fig. 9. The blank sheath of F2 was designed to delay the water molecules arriving at the core drug storage. Furthermore, the swelling performance of F2 could also be positively affected by the structural design, the outer layer swelled first and delayed the water invasion. Meantime, molecular gaps, left by drug molecules and dissolved PEG components, would be increased during the swelling process. For drug release, the increased molecular gaps after water invade swelling fibres weakened the protective structure of the drug stored in core layer, which accelerated the released rate. In the later stage II, drug molecules passed through the swelling blank sheath to the external release medium under the diffusion effects. However, more molecular gaps (after PEG dissolved) in F3 and F4 fibres could ‘absorb’ enough water molecules to invade fibres, which greatly weakened the role of the core–sheath structure in preventing water intrusion. This design of core–sheath fibres with a high PEG component accelerate the drug release to deal with the tailing–off effect.

The improvement of materials and structure brought the expected drug-release performance. The corporation of hydrophilic PEG improved the hydrophilicity of fibres and accelerated drugs release. Core–sheath structure provided a ‘barrier’ layer for drug reservoir to prolong the drug release. In this work, the ingenious combination of the two designs could not only improve the hydrophilic properties of fibres, but also greatly improved the sustained release performance. Simple preparation of functional materials by using the ‘one–pot’ modified triaxial electrospinning nanotechnology had a remarkable potential in pharmaceutics, drug delivery systems, and environment area. Based on the combination model of the structure and materials in this work, a considerable number of new strategies could be exploited, and applications were wide.

## Conclusion

In this work, we successfully prepared functional fibres with good hydrophilicity and sustained–release drugs by using modified triaxial electrospinning. The circulation of unspinnable solvent in the outer layer was beneficial to produce functional fibres during the modified electrospinning process. The design of functional F2–F4 fibres could be divided into two parts: (1) the additive of PEG improved the hydrophilicity, and (2) the core–shell structure brought the remarkable sustained–release drug properties. Although these designs led to the inhomogeneous and unexpected spindle morphology of fibres, XRD and FTIR results proved the amorphous form of the model drug and good compatibility between drug and CA polymeric matrix. In addition, the relative swelling properties of F2–F4 exhibited a similar and better water uptake rate compared with those observed in recent studies. Drug release data showed that functional F2 fibres provided a 96 h sustained-release drug profile, F3 and F4 fibres had a 12 h modified drug release profile by eliminating tailing–off phenomenon. Drug release mechanisms in F2–F4 were predominantly dominated by the Fickian diffusion. PEG component in functional F2–F4 fibres could form ‘passages’ to guide water molecules to invade the interior of fibres, and these ‘passages’ increased with the swelling of fibres. The dissolution of 1% PEG component had few negative effects on the drug release process, which contributed to the design of the core–shell structure. The blank-protected layer prolonged the time for water molecules to invade the inner core drug storage. However, the ‘barrier’ layer would lose its proper effect when the content of PEG over to 3%, but it could eliminate the inevitable tailing–off effect of late drug release.

Hydrophilic drug carriers and sustained-release drug cannot coexist traditionally. Fortunately, the combination of manipulating content of hydrophilic PEG and using the core–shell structure provided a good solution to solve the seemingly impossible challenges. The preparation of function F2–F4 fibres was an interdisciplinary field containing the advanced modified triaxial electrospinning and polymeric matrix with different physiochemical properties. Furthermore, the combination strategy of materials and structure in this work would inspire the production of more functional fibres.

## Supplementary Information

**Additional file 1: Figure S1.** Digital pictures of the modified coaxial electrospinning process of F1 monothetic fibres. **Figure S2.** The XRD pattern of different content of drugs in monothetic fibres. **Figure S3.** The surface wetting performance of different additives in CA monothetic fibres. **Figure S4.** SEM images of CA monothetic fibres containing hydrophilic additives before and after moisture immersion. **Figure S5.** The surface wetting performance of CA monothetic fibres containing 3% PEG **a** and 1% PEG. **Figure S6.** The dynamic surface wetting results of F1 fibres. **Figure S7.** Digital pictures of initial dry F1 (**a**), initial dry F2 (**b**), wet F1 (**c**), and wet F2 (**d**). **Figure S8.** Pictures of aluminum foil after the removal of F1 (**a**), F2 (**b**), F3 (**c**), and F4 (**d**) fibres.

## Data Availability

The datasets supporting the current study are available on reasonable request to the corresponding author.

## Abbreviations

CA: Cellulose acetate
PVP: Polyvinyl pyrrolidone
PEG: Polyethylene glycol
Cur: Curcumin
DMF: N, N-dimethylformamide
PBS: Phosphate buffer saline
SEM: Scanning electron microscope
TEM: Transmission electron microscope
FTIR: Fourier transformed infrared spectroscopy
XRD: X-ray diffraction
