# Supplementary material for: Elaborate design of shell component for manipulating the sustained release behavior from core–shell nanofibres
Source: J Nanobiotechnology. 2022 May 28;20:244. doi: 10.1186/s12951-022-01463-0 (PMC9148457; doi:10.1186/s12951-022-01463-0)
Supplement: Supplementary file 1 — Additional file 1: Figure S1. Digital pictures of the modified coaxial electrospinning process of F1 monothetic fibres. Figure S2. The XRD pattern of different content of drugs in monothetic fibres. Figure S3. The surface wetting performance of different additives in CA monothetic fibres. Figure S4. SEM images of CA monothetic fibres containing hydrophilic additives before and after moisture immersion. Figure S5. The surface wetting performance of CA monothetic fibres containing 3% PEG a and 1% PEG. Figure S6. The dynamic surface wetting results of F1 fibres. Figure S7. Digital pictures of initial dry F1 (a), initial dry F2 (b), wet F1 (c), and wet F2 (d). Figure S8. Pictures of aluminum foil after the removal of F1 (a), F2 (b), F3 (c), and F4 (d) fibres. [file 12951_2022_1463_MOESM1_ESM.docx]

**Supplementary Information**

**Elaborate design of shell component for manipulating the sustained release behavior from core–shell nanofibres**

Yubo Liu ^1†^, Xiaohong Chen ^12†^, Yuhang Gao ^1^, Deng-Guang Yu ^1,2^, Ping Liu ^1,2*^

^1^ School of Materials and Chemistry, University of Shanghai for Science & Technology, Shanghai 200093, China

^2^ Shanghai Engineering Technology Research Center for High-Performance Medical Device Materials, Shanghai 200093, China

†Yubo Liu and Xiaohong Chen contributed equally to this work

***Correspondence author**:

Prof. Ping Liu

School of Materials and Chemistry

University of Shanghai for Science and Technology

Address: 516 Jungong Road, Yangpu District, Shanghai 200093, China

E-mail: [pingliu_1962@163.com](mailto:pingliu_1962@163.com) (PL)

Tel: +86-021-55271656


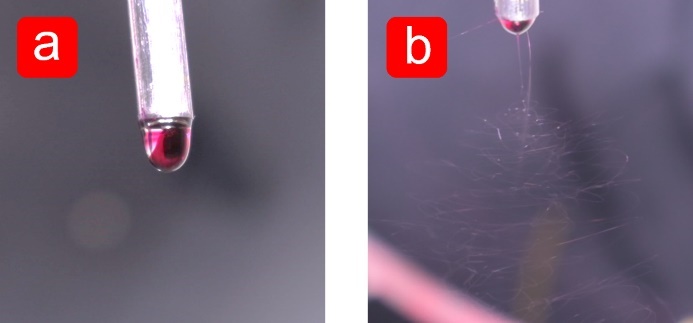


**Additional file 1: Figure S1.** Digital pictures of the modified coaxial electrospinning process of F1 monothetic fibres. (a) without voltage applied; (b) under the voltage of 9 kV.





**Additional file 1:** **Figure S2.** The XRD pattern of different content of drugs in monothetic fibres.


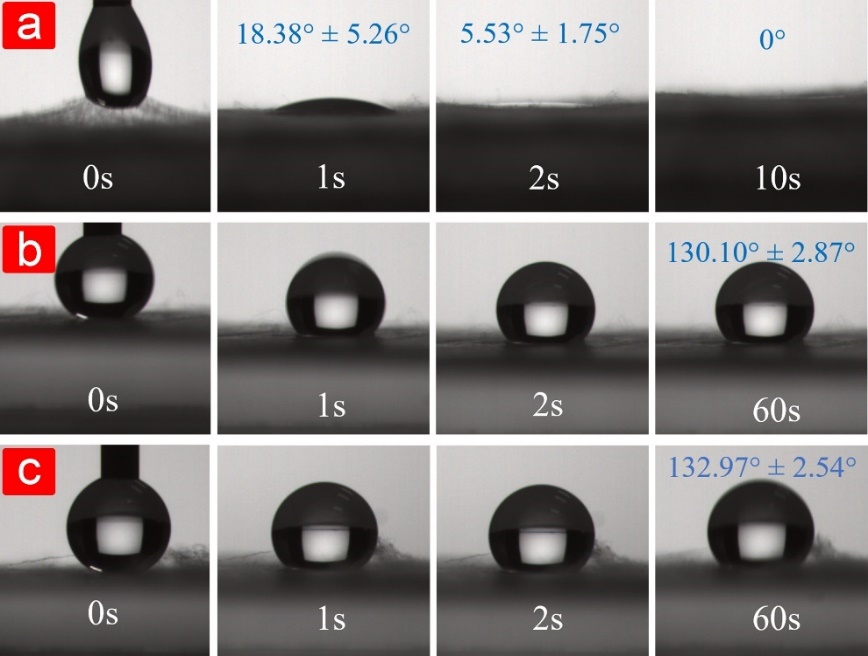


**Additional file 1:** **Figure S3.** The surface wetting performance of different additives in CA monothetic fibres. (a) 6% PEG; (b) 6% PVP K13-18; (c) 6% PVP K30


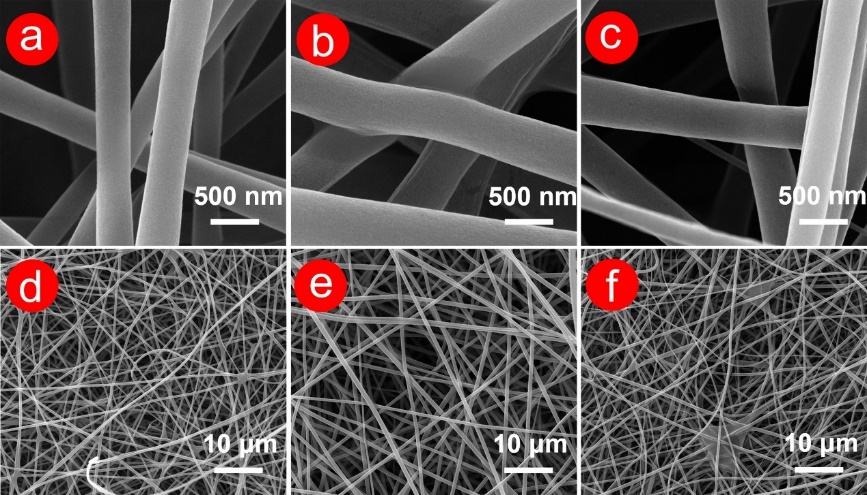


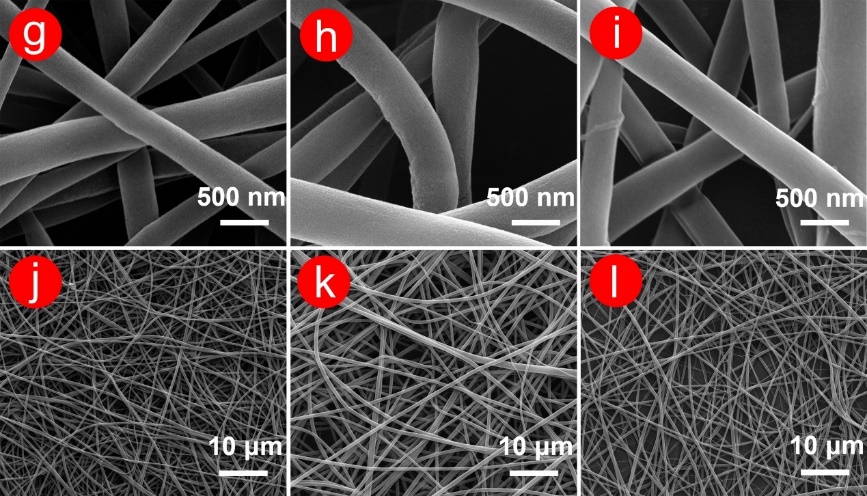


**Additional file 1:** **Figure S4.** SEM images of CA monothetic fibres containing hydrophilic additives before and after moisture immersion. Initial stage: (a) and (d) 15% CA + 6% PEG, (b) and (e) 15% CA + 6% PVP K13-18, (c) and (f) 15% CA + 6% PVP K30; Dry stage after soaking in water: (g) and (j) 15% CA + 6% PEG, (h) and (k) 15% CA + 6% PVP K13-18, (i) and (l) 15% CA + 6% PVP K30.


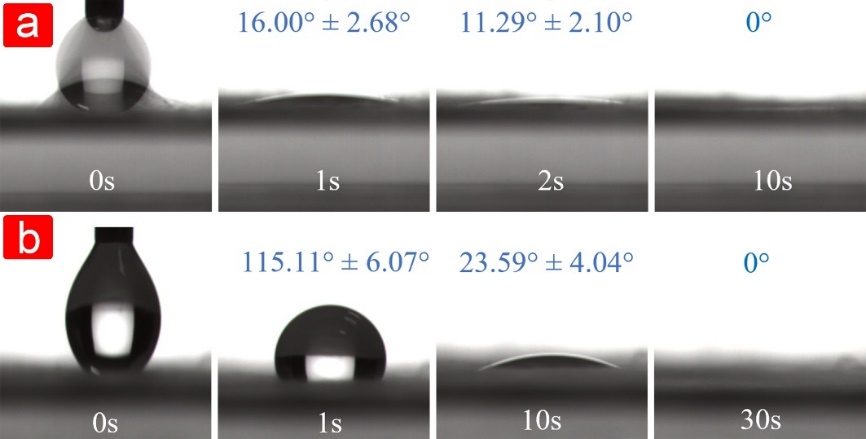


**Additional file 1:** **Figure S5.** The surface wetting performance of CA monothetic fibres containing 3% PEG (a) and 1% PEG.


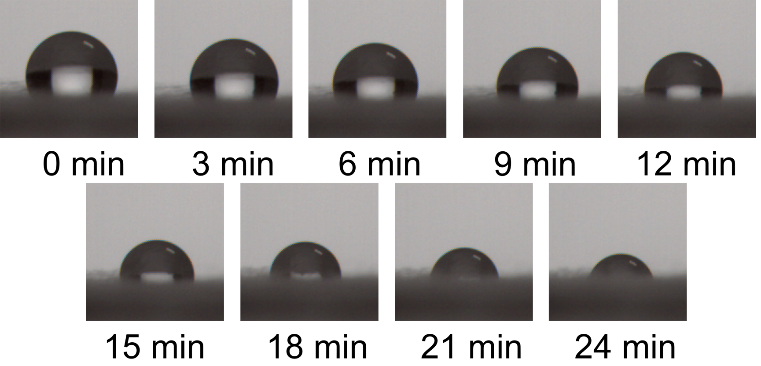


**Additional file 1:** **Figure S6.** The dynamic surface wetting results of F1 fibres.


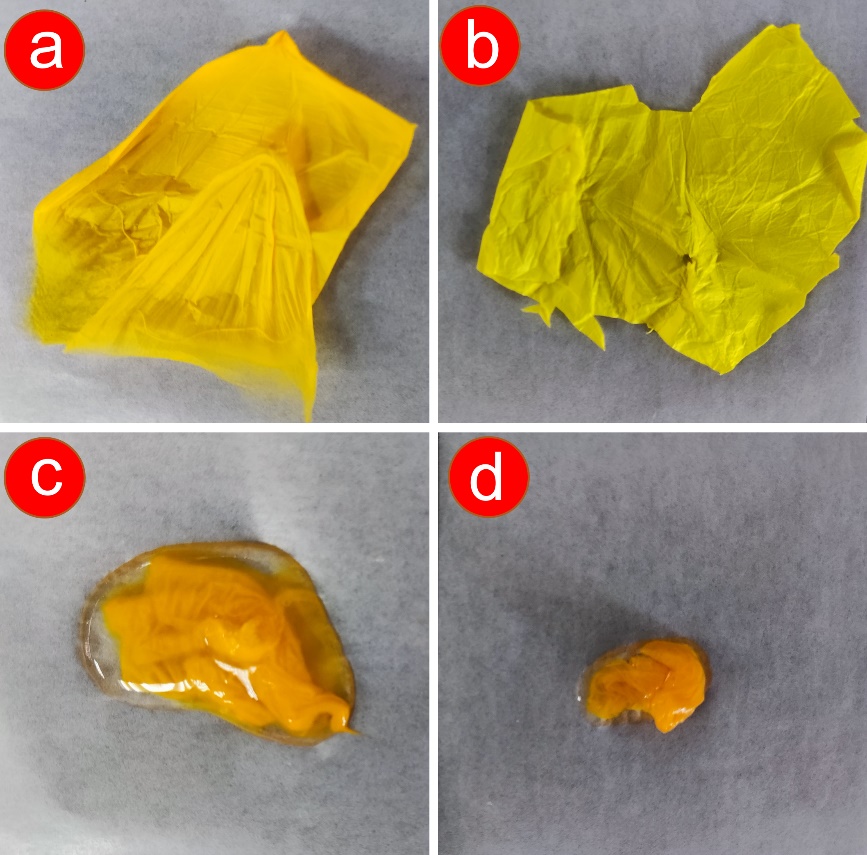


**Additional file 1: Figure S7.** Digital pictures of initial dry F1 (a), initial dry F2 (b), wet F1 (c), and wet F2 (d). F2 fibres were lighter in colour than F1 fibres due to the blank hydrophilic middle layer (CA and PEG). F1 and F2 shrank after fully absorbing water, especially F2, which looked only 1/10th of the original.


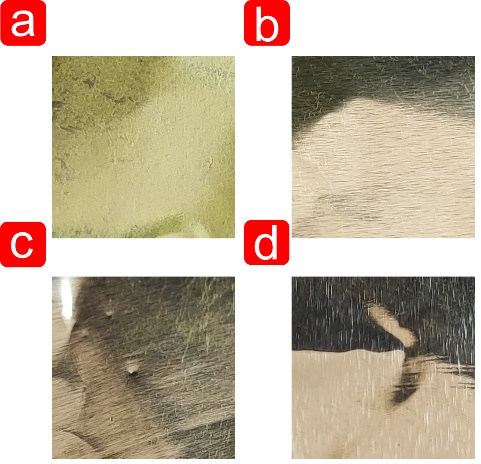


**Additional file 1:** **Figure S8.** Pictures of aluminum foil after the removal of F1 (a), F2 (b), F3 (c), and F4 (d) fibres.
